# Alveolar macrophages are sentinels of murine pulmonary homeostasis following inhaled antigen challenge

**DOI:** 10.1111/all.12536

**Published:** 2014-11-28

**Authors:** S A Mathie, K L Dixon, S A Walker, V Tyrrell, M Mondhe, V B O'Donnell, L G Gregory, C M Lloyd

**Affiliations:** 1 Leukocyte Biology, NHLI, Faculty of Medicine, Imperial College LondonLondon, UK; 2 UCB PharmaSlough, Berkshire, UK; 3 Department of Medical Biochemistry and Immunology, School of Medicine, Cardiff UniversityCardiff, UK

**Keywords:** Alveolar macrophage, homeostasis, house dust mite, interleukin-13, lung

## Abstract

**Background:**

Alveolar macrophages are sentinels of the pulmonary mucosa and central to maintaining immunological homeostasis. However, their role in governing the response to allergen is not fully understood. Inappropriate responses to the inhaled environment manifest as asthma.

**Methods:**

We utilized a mechanistic IL-13-driven model and a house dust mite allergen mucosal sensitization model of allergic airway disease to investigate the role of alveolar macrophages in regulating pulmonary inflammation.

**Results:**

IL-13-dependent eosinophilic and Th2 inflammation was enhanced in mice depleted of alveolar macrophages using clodronate liposomes. Similarly, depletion of alveolar macrophages during house dust mite sensitization or established disease resulted in augmented Th2 immunity and increased allergen-specific IgG1 and IgE. Clodronate treatment also delayed the resolution of tissue inflammation following cessation of allergen challenge. Strikingly, tissue interstitial macrophages were elevated in alveolar macrophage-deficient mice identifying a new homeostatic relationship between different macrophage subtypes. A novel role for the macrophage-derived immunoregulatory cytokine IL-27 was identified in modulating Th2 inflammation following mucosal allergen exposure.

**Conclusions:**

In summary, alveolar macrophages are critical regulators of Th2 immunity and their dysregulation promotes an inflammatory environment with exacerbation of allergen-induced airway pathology. Manipulating IL-27 may provide a novel therapeutic strategy for the treatment of asthma.

Allergic asthma is a chronic inflammatory disease which has traditionally been attributed to atopy, eosinophilia, and excessive Th2 mediators 1. However, clinical trials to target pro-inflammatory Th2 pathways have had limited success 2–4. Alveolar macrophages (AMs) are thought to play a central role in maintaining immunological homeostasis and host defense in the lung 5–8. Critical to their function, they are situated in the airway lumen, in close proximity to the mucosal surface where typically, they are the first immune cell to encounter the myriad particles that are contained within the inhaled environment. Human studies have demonstrated that the frequency of AMs in asthmatics and healthy individuals is comparable 9; however, some functionality may be altered in disease. AMs retrieved from asthmatics induce higher levels of IL-5 from T-lymphocyte co-cultures compared to those from nonatopic individuals 10. Additionally, AMs from pediatric asthmatics have impaired phagocytic ability and demonstrate greater apoptosis 11. In experimental systems, prior allergen exposure renders the lungs more susceptible to bacterial infection through desensitization of toll-like receptors (TLRs) and upregulation of endogenous negative regulators of TLR signalling expressed by AMs 12. Furthermore, GM-CSF, a mediator known to be upregulated in asthmatic bronchial epithelium 13, has been shown to inhibit the suppressive capacity of alveolar macrophages in animal studies 14,15. Thus, it is possible that the development of asthma, its lack of resolution, and associated exacerbations may be intimately linked with a breakdown in AM-mediated homeostasis.

Although inappropriate responses to airborne antigens are thought to contribute to the chronic inflammatory environment observed in asthma, the particular role that AMs play in controlling these responses is not well understood. Experimental studies utilizing rodents sensitized peripherally with OVA and the adjuvant alum prior to aerosolized OVA challenge have shown that depletion of AMs prior to challenge results in exacerbation of airway hyper-reactivity 16–19. However, AMs are resident within the airway mucosa and sample the inhaled environment; thus, peripheral sensitization models do not mimic how these cells encounter allergen in human disease. We have investigated the role of AMs at disease inception, during established inflammation and in promoting resolution of allergic airways disease (AAD) induced by repeated intranasal administration of house dust mite extract (HDM)—a clinically relevant allergen to which 50–85% of asthmatics are allergic to 20. We demonstrate that AMs are necessary to regulate mucosal sensitization to inhaled allergen. The macrophage-derived immune-regulatory cytokine IL-27 is implicated in maintaining pulmonary homeostasis. Moreover, evidence of a novel interaction between alveolar and interstitial macrophages was demonstrated. By identifying deficiencies in pulmonary defense and homeostatic mechanisms, we may be able to intervene to promote efficient resolution of inflammation and restore immune pulmonary homeostasis.

## Methods

Female BALB/c mice (Charles River, Bicester, UK) 6–8 weeks old received intratracheal administration of either IL-13 (5 μg; Gibco, Frederick, MD, USA) or vehicle (PBS + 1% FCS) on three consecutive days. Mice receiving liposomes were dosed 2 days prior to the first IL-13 administration and killed 15 h after the last administration. Additionally, mice received either 25 μg HDM extract *Dermatophagoides pteronyssinus* (Greer Laboratories, Lenoir, NC, USA) or PBS via intranasal instillation, 3 times per week for three weeks. Mice were killed either 4 h or 7 or 13 days postfinal HDM instillation. 50-μl liposome-encapsulated clodronate or PBS was administered intratracheally either once weekly, commencing 2 days prior to HDM exposure (sensitization phase) or twice during the final week of HDM exposure (challenge phase) or 1 and 5 days after the last HDM challenge (resolution phase). All mice were housed in specific pathogen-free conditions and provided food and water *ad libitum*. All experiments were performed in accordance with the UK Animals (Scientific Procedures) Act 1986.

Serum, bronchoalveolar lavage (BAL), lung tissue, and lung cells were collected. Differential counts were performed on Wright-Geimsa (Sigma, Poole, UK)-stained cytospins.

Disaggregated lung and BAL cells were stained with CD45, CD3, CD4, Siglec F, Gr-1, CD11c, CD11b (BD Biosciences, Oxford, UK), T1/ST2 (MD Biosciences, St. Paul, MN, USA), MOMA2, CD68 (AbD Serotec, Kidlington, UK) and F4/80 (eBiosciences, Hatfield, UK), or relevant isotype controls for 20 min at 4°C. Fixed cells were analyzed on a FACSCalibar™ using CellQuest (BD Biosciences).

Lung tissue supernatant and BAL were analyzed by ELISA; IL-4, IL-5 and IL-10 (PharMingen, Oxford, UK), IL-13 (eBioscience) and IL-27 (R&D systems, Abingdon, UK), or ultra-sensitive MSD kits (Meso Scale Discovery, Rockville, MD, USA). Paired antibodies for IgE and IgG1 (R&D systems) were used to measure serum Ig levels.

Tissue lipids were extracted, and lipoxin A4 was quantified by LC/MS/MS.

F4/80+ CD11c+ AMs and F4/80+ CD11c- interstitial macrophages were cell-sorted from lung tissue and BALF. RNA was extracted and IL-27 levels determined by qPCR using specific primers (Applied Biosystems, Paisley, UK).

Additional details on the methods utilized in this study are provided in the Supporting Information (Data S1).

Data were analyzed using Prism 5 (GraphPad Software Inc., La Jolla, CA, USA). Data are expressed as single data points with bars representing group medians, unless otherwise stated. A two-tailed *P* value was determined by the Mann–Whitney *U*-test when comparing between 2 groups. Data shown represent means ± SEM of at least 2 independent experiments (*n *=* *8–16).

## Results

### IL-13-induced Th2 airway pathology is amplified following the depletion of alveolar macrophages

**Figure 1:**
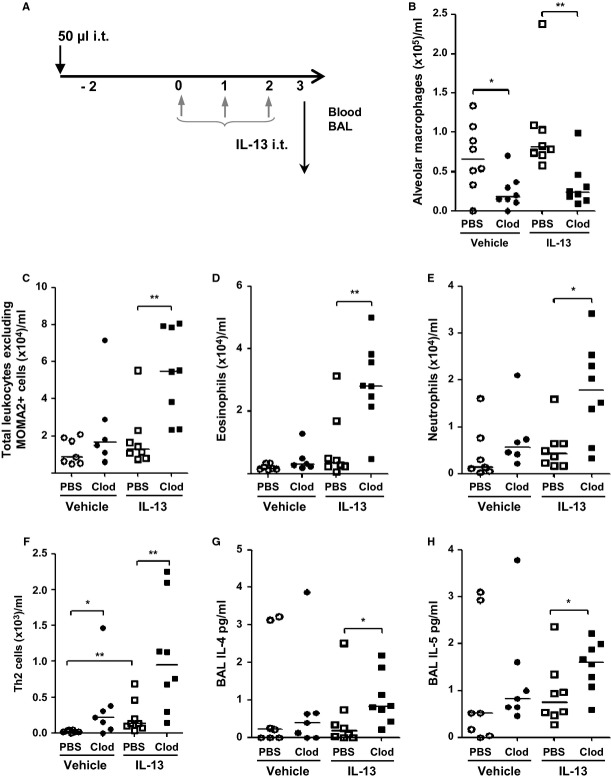
IL-13-induced Th2 airway pathology is amplified following the depletion of alveolar macrophages (A) Schematic depicting either PBS or clodronate (Clod) liposome and IL-13 administration protocol. Mice received either clodronate-containing liposomes (Clod) or PBS-containing liposomes (PBS). 48 hours later, mice received either 5 μg of IL-13 or PBS + 1% FCS (vehicle) for 3 consecutive days. Mice were culled 15 h later. (B) alveolar macrophages (C) total airway leukocytes (D) eosinophils (E) neutrophils (F) Th2 lymphocytes retrieved following broncho-alveolar lavage and quantified by flow cytometry. BAL fluid was analyzed for inflammatory cytokines by ELISA (G) IL-4 and (H) IL-5. *P < 0.05 and **P < 0.01 relative to PBS control group by Mann–Whitney *U*-test, *n* = 6–8.

To assess the role of AMs in the development of mucosal Th2 immunity, a short-term model of IL-13-induced lung inflammation was employed 21,22 in macrophage-replete mice and animals devoid of AMs. Clodronate liposomes administered 2 days prior to IL-13 instillation (Fig.1A) resulted in 80% depletion of AMs in BAL (Fig.1B), without affecting tissue-resident interstitial macrophages (Fig. S1). The proinflammatory effects of IL-13 were amplified in the absence of AMs, demonstrated by increased accumulation of eosinophils, neutrophils, and Th2 cells in BAL (Fig.1C–F). In addition, BAL IL-4 and IL-5 levels were elevated in AM-depleted mice (Fig.1G and H), although no enhancement of IL-10 was observed (Fig S2). These data suggest a protective role for AMs against development of Th2 immunity.

### The absence of alveolar macrophages augments HDM-induced airway Th2 inflammation

**Figure 2:**
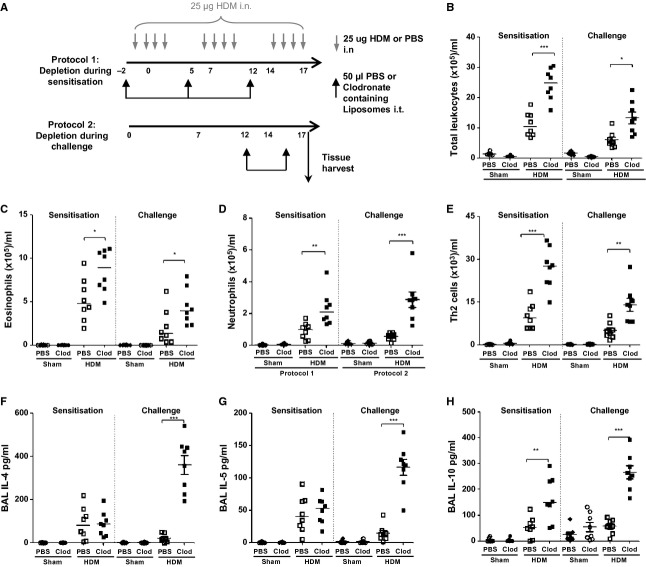
The absence of alveolar macrophages during mucosal allergen sensitization and challenge augments house dust mite-induced Th2 inflammation in the airways (A) Schematic depicting either PBS or clodronate (Clod) liposome and house dust mite (HDM) administration. (B) Total airway leukocytes, (C) eosinophils, (D) neutrophils, and (E) Th2 lymphocytes retrieved following bronchoalveolar lavage and quantified by flow cytometry. BAL fluid was analyzed for inflammatory cytokines by ELISA (F) IL-4, (G) IL-5, and (H) IL-10. *P < 0.05, **P < 0.01, and ***P < 0.001 relative to PBS control group by Mann–Whitney *U*-test, *n* = 6–8.

To explore the role of AMs during allergen sensitization and the development of AAD, clodronate was administered prior to HDM exposure and throughout the allergen sensitization period. AMs were also depleted during the final week of allergen challenge to investigate the role of AMs in perpetuating established AAD (Fig.2A).

House dust mite exposure increased accumulation of eosinophils, neutrophils and Th2 cells, and Th2 cytokines including IL-4, IL-5, and IL-10 in the BAL (Fig.2B–H). Depletion of AMs prior to allergen sensitization or during challenge significantly exacerbated HDM-induced lung inflammation. The number of BAL leukocytes in clodronate-treated mice was at least twofold greater than in HDM-exposed mice receiving control liposomes (Fig.2B). The absence of AMs resulted in increased numbers of both eosinophils and neutrophils (Fig.2C and D). Concomitant with the enhanced eosinophilia, eotaxin1/CCL11 was also increased in the BAL in the absence of AMs (Fig. S3A). In contrast, the neutrophil chemoattractant, KC/CXCL1, was only elevated when AMs were depleted during HDM challenge (Fig. S3B). In addition, AM deficiency during the sensitization or challenge phases significantly increased the number of Th2 cells in BAL (Fig.2E). However, exaggerated production of the Th2 cytokines IL-4 and IL-5 was only observed when AMs were depleted during allergen challenge (Fig.2F and G). No changes in HDM-induced IL-13 were detected (data not shown). Surprisingly, depletion of AMs during HDM sensitization or challenge further enhanced HDM-induced production of IL-10, IL-12, and IFNγ (Fig.2H and S3C and D). These findings indicate AMs are important for regulating the pulmonary immune response to inhaled allergen in the airways.

### Alveolar macrophage depletion influences the leukocyte composition of lung tissue and augments systemic immune responses following mucosal HDM exposure

**Figure 3:**
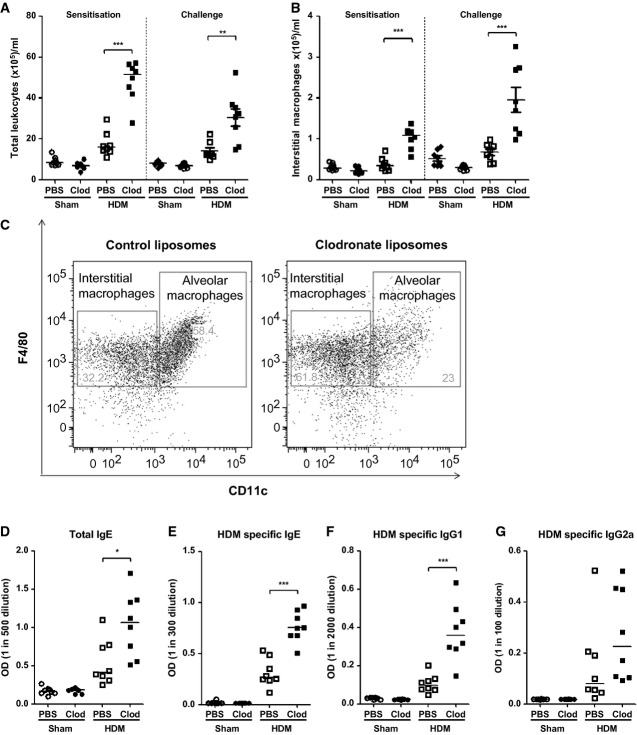
Disruption of alveolar macrophage-directed homeostasis promotes an increase in interstitial lung tissue macrophages and enhances the systemic immune response following HDM mucosal challenge (A) Total tissue leukocytes and (B) lung interstitial macrophages quantified by flow cytometry. (C) FACS plots depicting lung alveolar and interstitial macrophage populations. (D) Total IgE, (E) HDM-specific IgE, (F) HDM-specific IgG1, and (G) HDM-specific IgG2a in serum following clodronate treatment during sensitization quantified by ELISA. *P < 0.05, **P < 0.01, and ***P < 0.001 relative to PBS control group by Mann–Whitney *U*-test, *n* = 6–8.

Depletion of AMs during either allergen sensitization or challenge significantly increased the number of tissue leukocytes recruited to the lung in response to HDM exposure (Fig.3A). Tissue-resident interstitial macrophages have also been proposed to play a key role in maintaining immune homeostasis in the respiratory tract 23. Therefore, it was important to verify that this population of macrophages was not eliminated by the clodronate treatment. AM depletion actually increased the number of interstitial macrophages in response to HDM (Fig.3B and C), which was coincident with elevated levels of IL-10 (Fig.2H). Thus, the augmentation of Th2 airway pathology in HDM-exposed clodronate-treated mice is not due to the deletion of regulatory tissue interstitial macrophages.

The absence of AMs during HDM sensitization specifically enhanced the systemic Th2 antibody response. Total IgE and HDM-specific IgE and IgG1 were significantly elevated in HDM-exposed mice pretreated with clodronate liposomes (Fig.3D–F), whereas HDM-specific IgG2a was not modulated (Fig.3G). The absence of AMs in the respiratory tract has far-reaching consequences both in the lung parenchyma and on systemic Th2 responses following allergen exposure.

### Alveolar macrophages are elevated concomitant with the resolution of HDM-induced inflammation

**Figure 4:**
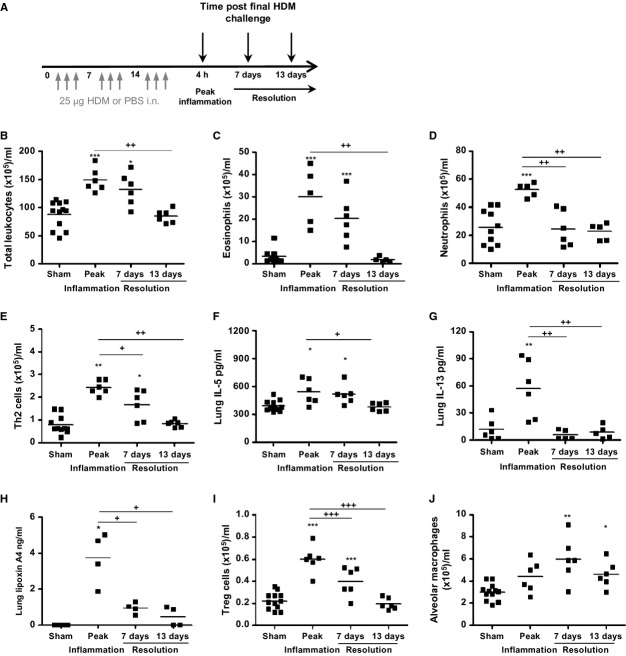
Alveolar macrophages are elevated concomitant with the resolution of mucosal inflammation (A) Schematic depicting PBS or HDM, (B) total tissue leukocytes, (C) eosinophils, and (D) neutrophils were quantified by differential counting. (E) Th2 lymphocytes quantified by flow cytometry. Th2 cytokine levels in lung tissue homogenate were quantified by ELISA (F) IL-5 and (G) IL-13. (H) LC/MS/MS was used to quantify lung tissue lipoxin A4. (I) CD4^+^CD25^+^FoxP3^+^ T-regulatory lymphocytes and (J) alveolar macrophages were quantified by flow cytometry. At each time point *n* = 4–12 mice/group. *P < 0.05, **P < 0.01, and ***P < 0.001 relative to PBS control group by Mann–Whitney *U*-test. ^+^*P* < 0.05, ^++^*P* < 0.01, and ^+++^*P* < 0.001 relative to peak inflammation, 4-h time point group by Mann–Whitney *U*-test.

We next sought to ascertain whether AMs play an active role in the resolution of HDM-induced AAD. Mediators of allergic disease were measured 7 and 13 days after the last allergen challenge and compared to peak inflammation, 4 h post-HDM (Fig.4A). HDM-induced inflammation remained significantly elevated 7 days postfinal challenge and resolved to baseline levels by 13 days (Figs4B and S4A). Tissue eosinophils remained at least threefold higher at 7 days, resolving by 13 days (Fig.4C). In the BAL, inflammation was also predominantly eosinophilic. Numbers were significantly reduced by 13 days, but some residual eosinophils remained (Fig. S4B). HDM-induced neutrophilia, evident at peak inflammation, resolved more rapidly and returned to baseline by 7 days (Figs4D and S4C). HDM-induced Th2 lymphocytes and IL-5 and IL-13 levels remained elevated 7 days post-HDM and resolved by day 13 (Fig.4E–G). Lipoxin A4 was induced following HDM exposure, and elevated levels were measured throughout the resolution time course indicating that lipoxin A4 is involved in the active resolution of inflammation (Fig.4H). FoxP3^+^ T-regulatory cells remained significantly elevated at 7 days and returned to baseline levels by 13 days after the final allergen exposure (Fig.4I). AMs were significantly elevated following cessation of allergen challenge (Fig.4J) implying that these cells play an active role in directing the return to pulmonary homeostasis.

### Return to homeostasis is delayed following the removal of alveolar macrophages

**Figure 5:**
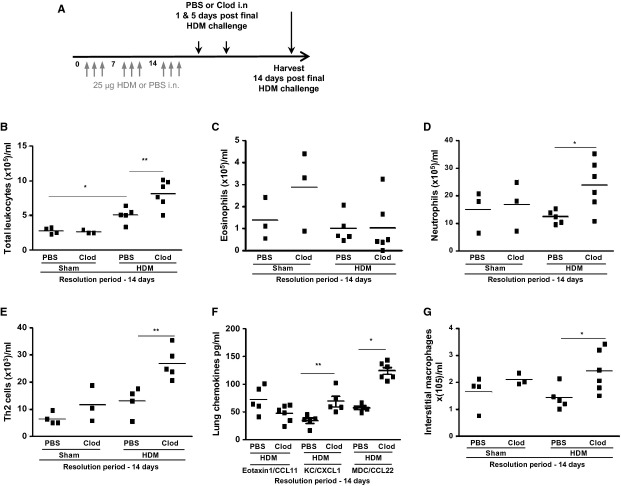
The return to homeostasis after allergen challenge is disrupted following the removal of alveolar macrophages (A) Schematic depicting PBS or clodronate (Clod) liposome administration protocol following the induction of allergic airways disease. (B) airway leukocytes retrieved following broncho-alveolar lavage and (C) tissue eosinophils; (D) tissue neutrophils determined by differential counting. (E) Lung tissue Th2 lymphocytes quantified by flow cytometry. (F) lung tissue chemokines measured by ELISA. (G) interstitial macrophages determined by flow cytometry, *n* = 3–6 mice/group, bar represents median. *P < 0.05 and **P < 0.01 relative to PBS control group by Mann–Whitney *U*-test, *n* = 3–6.

Alveolar macrophages were depleted during the resolution phase (Fig.5A), and parameters of airway inflammation were assessed 14 days postfinal HDM exposure. Administration of clodronate liposomes during the resolution phase prevented the clearance of HDM-induced airway inflammatory cells (Fig.5B). Eosinophilia, which resolves by day 14, was not altered by macrophage depletion (Fig.5C); however, HDM-induced neutrophilia and Th2 cells failed to resolve in the absence of AMs (Fig.5D and E). No change in the profile of IL-4, IL-5, or IL-13 levels was observed as a result of depleting AMs during resolution (data not shown). However, levels of KC/CXCL1 and MDC/CCL22 remained elevated at 14 days correlating with the neutrophil and T-cell numbers in clodronate-treated mice (Fig.5F), whereas eotaxin1/CCL11 levels returned to baseline with resolution of eosinophilia.

The interstitial macrophage population was not modulated by exposure to HDM or AM depletion alone; however, numbers of pulmonary interstitial macrophages were increased in HDM-exposed mice treated with clodronate during the resolution phase. (Fig.5G).

### Exacerbation of HDM-induced disease is associated with reduced levels of IL-27

**Figure 6:**
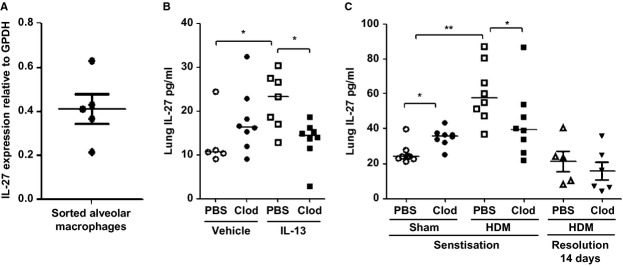
Exacerbation of IL-13- and HDM-induced disease following alveolar macrophage depletion is associated with reduced levels of the immuno-regulatory cytokine IL-27. IL-27 mRNA expression in lung FACS-sorted macrophages analyzed by qPCR (A) F4/80^+^ CD11c+ alveolar macrophages, (B) IL-13-treated mice, and (C) HDM-treated mice with alveolar macrophage depletion during sensitization and during resolution *P < 0.05 and **P < 0.01 relative to PBS control group by Mann–Whitney *U*-test, *n* = 5–8.

We sought to determine whether immunoregulatory mediators were modulated as a consequence of macrophage depletion. TGF-β was not significantly altered following alveolar macrophage manipulation (data not shown), and we demonstrated elevated IL-10 levels (Fig.2H). Thus, we hypothesized that there may be a role for the myeloid-derived, immunoregulatory cytokine IL-27 in our models. To confirm AMs were a source of IL-27, we analyzed FACS-sorted cells and demonstrated the expression of IL-27 in this population (Fig.6A).

In the lung tissue, levels of IL-27 were upregulated in response to IL-13 or repeated HDM exposure (Fig.6B and C). Interestingly, the depletion of AMs also increased baseline levels of IL-27 in nonallergic mice. However, clodronate treatment during the sensitization phase resulted in a reduction of HDM-induced IL-27 concomitant with exacerbation of Th2 pathology. Depletion of AMs during the resolution phase did not modulate tissue levels of IL-27 which return to baseline by 14 days. These data demonstrate that AMs are a source of IL-27 and suggest a specific temporal role for IL-27 in regulating Th2 responses at mucosal surfaces.

## Discussion

This work characterizes for the first time the immunomodulatory role of AMs in a model of inhaled allergen-induced AAD. It is evident that AMs are critical for the maintenance of pulmonary homeostasis and disruption of this axis results in uncontrolled Th2 inflammation in response to HDM and IL-13. We also show that AMs drive the resolution of inflammation following allergen challenge. In healthy individuals, the primary role of AMs is in maintaining or restoring homeostasis; however, in asthmatics, inappropriate or insufficient AM activity may perpetuate chronic inflammation by lowering the immune activation threshold.

Activation of mucosal epithelial cells in response to inhaled particulates can lead to the infiltration of granulocytes, which release harmful cytotoxic mediators intended to protect host tissues; however, left unchecked, these are potentially deleterious to the local microenvironment 24. Thus, granulocyte accumulation warrants tight regulation to preserve tissue integrity 25,26. Signals from apoptotic granulocytes induce their efferocytosis by local tissue macrophages 26, and our data show that macrophages are essential to limit the accumulation of eosinophils and neutrophils in response to HDM exposure, promoting a return to tissue homeostasis.

House dust mite-challenged mice-lacking AMs during the sensitization phase had increased Th2 cell recruitment compared to macrophage-replete mice. When clodronate was administered during the HDM challenge phase, once inflammation was established, Th2 immunity was also augmented, with increased accumulation of Th2 cells within the airways and elevated IL-4 and IL-5. Cytokines were quantified 5 days and 24 h after the last clodronate treatment in the sensitization and challenge protocols, respectively. Thus, the dysregulation of Th2 cytokines following AM depletion appears to be transient and wanes as AMs begin to repopulate the airways. Our data corroborate the studies utilizing the OVA model in which augmented airway pathology was observed following AM removal 17–19. However, our work further demonstrates that AMs constrain granulocyte and effector T-cell accumulation in response to inhaled allergen exposure irrespective of prior sensitization. In addition, we have established a critical role for AMs during the different stages of disease pathogenesis.

Our data demonstrate that the immune activation threshold is reduced in the absence of AMs and that these cells limit HDM-induced allergic inflammation during established disease suggesting that AMs maintain a regulatory phenotype throughout acute HDM exposure. In contrast, adoptive transfer of AMs from OVA-sensitized mice fails to restore homeostasis compared to transfer of naive AMs 19. It was proposed that OVA sensitization and challenge altered the phenotype of AMs to produce inflammatory cytokines and lose their suppressive capacity. However, *ex vivo* culture of allergen-sensitized AMs were shown to downregulate their pro-inflammatory phenotype and restore their homeostatic functions when subsequently transferred 27,28. This exploitation of macrophage plasticity makes these cells an interesting potential target for therapy 29.

We investigated the role of AMs in promoting resolution and restoring homeostasis by depleting AMs after cessation of HDM exposure. This resulted in delayed resolution of Th2 immunity, indicating that AMs are involved in the clearance of T-cell infiltrates recruited to the lung as a result of allergen exposure.

Augmented inflammation was apparent within the lung tissue and airway lumen as a result of the loss of AM-directed homeostasis. Perhaps unexpectedly, the number of interstitial macrophages in lung tissue was significantly increased during exacerbated AAD. This expansion of interstitial macrophages following the depletion of AMs was apparent in all three HDM protocols indicating a direct relationship between these two regulatory macrophage cell types. Interstitial macrophages have been reported to regulate airway inflammation via TLR4-dependent IL-10 secretion 23. Additionally, interstitial macrophages have been shown to express high levels of eotaxin1/CCL11 30. HDM extract contains TLR4 agonists 31, and enhanced levels of IL-10 and eotaxin1/CCL11 were apparent in our studies concomitant with the increased number of interstitial macrophages. This suggests that interstitial macrophage functionality remains intact during exacerbated AAD and that the regulation of this subset is impaired in the absence of AMs. The role of interstitial macrophages remains unclear in the context of asthma. Production of eotaxin1/CCL11 and IL-10 suggests they have the potential to exert a dual role in an allergic setting and further investigation is required. From our studies, we clearly demonstrate that an important axis exists between airway and tissue macrophage subsets.

The potent effects of removing AMs led us to investigate potential mediators responsible for this dysregulated homeostasis. IL-27 is predominantly produced by macrophages and dendritic cells. This cytokine is upregulated in steroid refractory asthmatics 32 and has been shown to modulate airway hyper-reactivity via downregulation of glucocorticoid receptor signalling in macrophages 32. IL-27 also dampens Th2 cell polarization and cytokine production in naïve CD4^+^ T-cell cultures and ameliorates OVA-induced allergic inflammation when administered *in vivo* 33. In the gut, IL-27 has been shown to mediate intestinal epithelial cell barrier protection via transcriptional activation of anti-inflammatory and antibacterial pathways 34. We demonstrate for the first time that IL-27 is upregulated in the lung following the administration of either exogenous IL-13 or HDM and that AMs are a source of this cytokine. These data demonstrate that IL-27 is a macrophage-derived immune-regulatory mediator which regulates the pulmonary mucosal response to allergen.

In summary, we show that AMs are integral in the regulation of inflammatory responses to inhaled allergen. We also show for the first time a direct relationship between airway alveolar and tissue interstitial macrophage populations. Thus, macrophages play a critical role in the maintenance and restoration of homeostasis following allergen exposure at the airway mucosa.

## Supporting Information

**Figure S1.**Alveolar macrophages are depleted following clodronate treatment. **Figure S2.** IL-10 levels are not modulated in response to IL-13 and are unaffected by macrophage depletion. **Figure S3.** Alveolar macrophage depletion augments house dust mite induced chemokine and cytokine secretion. **Figure S4.** Resolution of inflammation following cessation of allergen challenge.
