# Supplementary material for: Alveolar macrophages are sentinels of murine pulmonary homeostasis following inhaled antigen challenge
Source: Allergy. 2014 Nov 28;70(1):80–9. doi: 10.1111/all.12536 (PMC4283732; doi:10.1111/all.12536)
Supplement: Supplementary file 2 — Data S1. Materials and methods. [file all0070-0080-sd2.docx]

Supplementary data

Alveolar macrophages are sentinels of murine pulmonary homeostasis following inhaled antigen challenge.

Sara A. Mathie, Kate L. Dixon, Simone A. Walker, Victoria Tyrrell, Madhav Mondhe, Valerie B. O’Donnell, Lisa G. Gregory, & Clare M. Lloyd

Materials and Methods

**Measurement of AHR**

Measurements of dynamic resistance and compliance were performed using The Flexivent system (Scireq, Montreal, Canada). After induction of anaesthesia with an i.p. injection of Pentobarbitone (50 mg/Kg) (Sigma, UK) and i.m. injection of Ketamine (200 mg/Kg) (Fortdodge Animal Health Ltd, Southampton, UK), mice were tracheostomised and connected to the Flexivent ventilator via a blunt-ended 19-gauge needle. Mice were ventilated using the following settings; tidal volume of 7 ml/kg body weight, 150 breaths/minute; positive end-expiratory pressure approximately 2 cm H_2_O. Standardisation of the lung volume history was done by performing two deep inflations. Subsequently, measurements of airway function were made. Measurements of resistance and compliance were determined from a user defined protocol using the snapshot-150 perturbation, which is a single frequency sinusoidal waveform. Resultant data was fitted using multiple linear regression to the single compartment model to determine the above parameters. The resultant data was fitted to the Constant-Phase model to determine the above parameters. Changes in lung/airway function parameters were measured in response to increasing concentrations of nebulised MCh from 3 - 100 mg/ml, delivered into the inspiratory line of the flexivent ventilator. Airway resistance was expressed as cmH_2_O.s/ml, elastance was expressed as cmH2O/mL and compliance was expressed as mL/cmH_2_O.

**Collection and preparation of samples**

Bronchoalveolar lavage (BAL) was collected by lavage of the lungs three times with 0.4 mL of PBS via a tracheal cannula. BAL fluid was centrifuged (700 *g*, 5 min, 4°C) and cells were recovered and resuspended in 0.5ml RPMI. BAL cell supernatants were removed and analyzed for cytokines by ELISA. To disaggregate the cells from the lung tissue, one lobe (
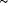
100 mg) of lung was incubated at 37°C for 1 h in digest reagent (0.15 mg/ml collagenase type D, 25 µg/ml DNase type I) in complete RPMI media. The recovered cells were filtered through a 70-µm nylon sieve, washed twice, resuspended in 1ml complete media, and counted in a hemocytometer prior to cytocentrifugation. Lung and BAL cells were applied to glass slides by centrifugation (5x10^4^ cells/slide) and stained with Wright-Giemsa (Thermo Fisher Scientific Inc, Waltham, MA). Percentages of macrophages, lymphocytes/mononuclear cells, eosinophils and neutrophils were determined under 40x magnification by counting cells in 8 randomly selected fields and dividing this number by the total number of cells counted. To obtain absolute numbers, this percentage was multiplied by the total number of cells recovered in 1 ml of lavage fluid and lung digest suspension which were normalised for the weight of the lung.

**Lipid extraction and quantitation**

For quantification, 10ng of each 12-HETE-*d_8_* and PGE2-*d_4_* was added to BALF or lung homogenate before extraction as deuterated internal standards. Lipids were extracted by adding a solvent mixture [1 M acetic acid, 2-propanol, hexane (2:20:30, v/v/v)] to the sample at a ratio of 2.5mL solvent mixture/1mL sample, vortexing and then adding 2.5 mL hexane. After centrifugation, lipids were recovered in the upper hexane layer. The samples were then re-extracted by addition of an equal volume of hexane. The combined hexane layers were dried under vacuum and analyzed for free eicosanoids using liquid chromatography-coupled to electrospray ionisation tandem mass spectrometry (LC-MS/MS ABI SCIEX 4000 QTRAP) as follows: Lipids were separated on a C_18_ Spherisorb ODS2 column (150 x 4.6 mm, 5µm particle, Waters Ltd, Elstree, UK) using a gradient of 50–90% B over 30 min, followed by 5 min at 90% B (A, water : acetonitrile : acetic acid, 75 : 25 : 0.1, B, methanol : acetonitrile : acetic acid, 60 : 40 : 0.1) with a flow rate of 1 mL/min. Eicosanoid species were monitored with specific parent to daughter ion transitions in negative ion mode ([M−H]^−^) for LXA4 (*m/z* 351.2) at 115. 12-HETE-*d_8_* and PGE_2_-*d_4_* was monitored at *m/z* 327 to 184 and *m/z* 335.2 to 275 respectively. Products were identified and quantified using primary standards and internal standard run in parallel under the same method conditions.

**Macrophage cell sort**

Whole lung was digested as previously described. Isolated leukocytes were incubated with FITC conjugated CCR3 and Ly6C, F4/80 (PeCY7), CD11c (PE) and CD11b (APC). Cells were negatively selected for CCR3 and Ly6C to remove eosinophils and neutrophils. F4/80+ cells were sorted for CD11c and CD11b expression. Alveolar macrophages were defined as CD11c+ and IMs as CD11c-. Cells were sorted into RLT buffer for RNA extraction.

**Quantitative PCR**

Quantitative PCR was performed on FACS sorted macrophages. Briefly, RNA was prepared from cell isolated as per manufacturer’s instructions (Qiagen). cDNA was synthesised using oligo(dT)-primed reverse transcriptase reactions. (Applied Biosystems). Quantitative PCR was performed using taqman probes for IL-27 and GAPDH. Levels of IL-27 mRNA were normalised to GAPDH.

**Figure Legends**

**Figure S1. Alveolar macrophages are depleted following clodronate treatment**

Mice received either clodronate containing liposomes (Clod) or PBS containing liposomes (PBS). (A) Alveolar macrophage and (B) Interstitial macrophages numbers 1, 4 and 7 days post PBS or Clod administration quantified by flow cytometry. * p<0.05 and ** p<0.01 relative to PBS control group by Mann Whitney test, n=4-5.

**Figure S2. IL-10 levels are not modulated in response to IL-13 and are unaffected by macrophage depletion**

BAL fluid was analysed for IL-10 by ELISA. n=7-8.

**Figure S3. Alveolar macrophage depletion augments house dust mite induced chemokine and cytokine secretion**

BAL fluid was analysed for inflammatory cytokines (A) eotaxin, (B) KC, (C) IL-12 and (D) IFN-γ by ELISA. * p<0.05 and *** p<0.001 relative to PBS control group by Mann Whitney test, n=5-8.

**Figure S4. Resolution of inflammation following cessation of allergen challenge**

(A) Total leukocytes (B) eosinophils and (C) neutrophils recovered from the BALF were quantified by differential counting. ** p<0.01 and *** p<0.001 relative to PBS control group by Mann Whitney test. ^+^ p<0.05 and ^++^p<0.01 relative to peak inflammation, 4 hour time point group by Mann Whitney test, n=5-8.
